# Anti-Inflammatory Activity and Changes in Antioxidant Properties of Leaf and Stem Extracts from *Vitex mollis* Kunth during *In Vitro* Digestion

**DOI:** 10.1155/2015/349235

**Published:** 2015-09-15

**Authors:** Juan Alfredo Morales-Del-Rio, Melesio Gutiérrez-Lomelí, Miguel Angel Robles-García, Jose Antonio Aguilar, Eugenia Lugo-Cervantes, Pedro Javier Guerrero-Medina, Saul Ruiz-Cruz, Francisco J. Cinco-Moroyoqui, Francisco J. Wong-Corral, Carmen Lizette Del-Toro-Sánchez

**Affiliations:** ^1^División de Desarrollo Biotecnológico, Centro Universitario de la Ciénega (CUCI), Universidad de Guadalajara (UdeG), Avenida Universidad 1115, Colonia Lindavista, 47820 Ocotlán, JAL, Mexico; ^2^Coordinación de Genómica Alimentaria, Universidad de la Ciénega del Estado de Michoacán de Ocampo (UCM), Avenida Universidad 3000, Colonia Lomas de la Universidad, 59103 Sahuayo, MICH, Mexico; ^3^Unidad de Tecnología Alimentaria, Centro de Investigación y Asistencia en Tecnología y Diseño del Estado de Jalisco (CIATEJ) AC, Avenida Normalistas 800, Colonia Colinas de la Normal, 44270 Guadalajara, JAL, Mexico; ^4^Departamento de Biotecnología y Ciencias Alimentarias, Instituto Tecnológico de Sonora (ITSON), 5 de Febrero 818 Sur, Colonia Centro, 85000 Ciudad Obregón, SON, Mexico; ^5^Departamento de Investigación y Posgrado en Alimentos, Universidad de Sonora, Rosales y Niños Héroes S/N, 83000 Hermosillo, SON, Mexico

## Abstract

*Vitex mollis* is used in traditional Mexican medicine for the treatment of some ailments. However, there are no studies on what happens to the anti-inflammatory activity or antioxidant properties and total phenolic content of leaves and stem extracts of *Vitex mollis* during the digestion process; hence, this is the aim of this work. Methanolic, acetonic, and hexanic extracts were obtained from both parts of the plant. Extract yields and anti-inflammatory activity (elastase inhibition) were measured. Additionally, changes in antioxidant activity (DPPH and ABTS) and total phenols content of plant extracts before and after *in vitro* digestion were determined. The highest elastase inhibition to prevent inflammation was presented by hexanic extracts (leaf = 94.63% and stem = 98.30%). On the other hand, the major extract yield (16.14%), antioxidant properties (ABTS = 98.51% and DPPH = 94.47% of inhibition), and total phenols (33.70 mg GAE/g of dried sample) were showed by leaf methanolic extract. Finally, leaf and stem methanolic extracts presented an antioxidant activity increase of 35.25% and 27.22%, respectively, in comparison to their initial values after *in vitro* digestion process. All samples showed a decrease in total phenols at the end of the digestion. These results could be the basis to search for new therapeutic agents from *Vitex mollis*.

## 1. Introduction

Inflammation is a complex process and is a protective reaction of cells/tissues of the body to allergic or chemical irritation, injury, and/or infections [1]. This process has many ways to act; one of them is by elastase enzyme [EC: 3.4.4.7]. This enzyme is a serine protease associated with the granular fraction of polymorphonuclear leukocytes, which hydrolyzes multiple bonds of connective tissue matrix protein substrates such as elastin, collagen, proteoglycans, and keratins [2]. UV irradiation stimulates the activity of fibroblast elastases in skin [3]. Additionally, elastase can display chronic obstructive pulmonary disease-like features including widespread lung inflammation, goblet cell metaplasia, increased lung volume, emphysema, and decreased elastic recoil [4]. Therefore, elastase plays a critical role in inflammatory processes. Plants with potent biological effect and fewer side effects have attracted great interest and have been evaluated for their use in treating inflammation [2, 5–7].

The use of plants is becoming more important not only for their anti-inflammatory properties but also for their ability to prevent cellular damage induced by free radicals. The excess of free radicals is produced during oxidative metabolism causing damage in cellular lipids, proteins, or DNA, inhibiting their normal function [8]. For these reasons, the importance of free radicals neutralization by antioxidants is a very important aspect of living organisms. Phenolic compounds are commonly found in plants exhibiting antioxidant properties that are attributed to their intrinsic reducing properties [9]. However, when the plants or their extracts are consumed, the digestive process can affect their antioxidant properties due to their hydrophobicity, molecular mass, degree of polymerization, environment, kind of sugar present in the molecule, and pH [10]. Furthermore, several studies have shown that the effectiveness of phenols absorption through the intestine is influenced by the action of digestive enzymes such as pepsin and pancreatin [11, 12]. Therefore, the importance of the antioxidant properties of plant materials in the maintenance of health and protection against several diseases has raised interest among scientists [13]. In this context,* Vitex mollis* is a medicinal plant widely used in traditional Mexican medicine for the treatment of diarrhea and dysentery [14] that possesses antispasmodic [14], antiprotozoal [15], and antioxidant [16] activities. The aim of the present work was to investigate the anti-inflammatory properties of leaves and stem extracts of* Vitex mollis*, as well as changes in their total phenol contents and antioxidant properties that might occur during the digestion process.

## 2. Methodology

The material and reagents such as porcine pancreatic elastase (PPE, type IV), succinyl-Ala-Ala-Ala-*p*-nitroanilide (ESIV, elastase substrate IV), DPPH [2,2-diphenyl-1-picrylhydrazyl], ABTS [2,2′-azino-bis-(3-ethylbenzothiazoline-6-sulfonic acid)], gallic acid [3,4,5-trihydroxybenzoic acid], 2 N Folin-Ciocalteu phenol reagent solution, tris-HCl buffer (T-3253), pepsin (P-7012), pancreatin (P-1750), phosphate buffered saline (PBS-3813), and dialysis tubing (D-9527) were purchased from Sigma-Aldrich Co. (USA). All other chemicals and solvents were of the highest commercial grade.

### 2.1. Extraction

Leaves and stems of* Vitex mollis* were collected from the Ciénega region of Jalisco, Mexico (102°48′W, 20°21.6′N, 1800 m of altitude), in May 2014. Taxonomic identification of the plant was made by the Botanic Institute of the University of Guadalajara (IBUG) with the register number 192156. The extractions of compounds were made according to Del-Toro-Sánchez et al. [17] with some modifications. Three grams of each dried and ground sample was mixed with 20 mL of solvent (methanol, acetone, or hexane). After homogenization for 1 min (Ultraturrax, T 25 DS1 digital homogenizer) and sonication at 4°C for 15 min (Bransonic, 151-DTH), the samples were centrifuged at 4,000 ×g for 15 min at 4°C. The supernatants were collected and the residues were used for a second extraction employing the same procedure. The supernatants were combined and evaporated to dryness using a rotavapor (Heidolph Rotavapor, 4003 VAC Senso T) at 45–50°C. The dry extracts were weighed to obtain the yield of the extract and solubilized in aqueous 1% Tween 20 to a final concentration of 750 *μ*g/mL. Leaves samples were identified as methanolic, acetonic, and hexanic extracts (LM, LA, and LH, resp.). The same designation procedure was used for the stem extracts (SM, SA, and SH).

### 2.2. Anti-Inflammatory Activity

The anti-inflammatory activity was evaluated by the method of Lee et al. [2]. This method consists in the spectrophotometric measurement at 410 nm of the amount of* p*-nitroaniline released from the substrate [*N*-Succ-(Ala)3-*p*-nitroanilide] by porcine pancreatic elastase (PPE). The reaction mixture (final volume of 150 *μ*L) contained 0.2 M Tris-HCl buffer (pH 8.0), 1 *μ*g/mL elastase (0.046 U/mL), 0.8 mM N-succinyl-Ala-Ala-Ala-*p*-nitroanilide as substrate, and leaves and stem extracts as inhibitors. Aliquots (10 *μ*L) of the extracts, previously incubated for 20 min at 25°C, were added at different concentrations (0, 150, 300, 450, 600, and 750 *μ*g/mL). The reaction was started by adding the substrate. Proper blanks were prepared and contained all the components except the enzyme. The rate of the reaction was determined as the slope of the line recorded and was proportional to elastase activity. A control curve was prepared with elastase in the absence of inhibitor. One unit of elastolytic activity was defined as the amount of enzyme required to produce 1 mM of* p*-nitroaniline/min under the conditions of the assay. For* p*-nitroaniline, an *ε* of 8,800 at 410 nm was employed. Percentage of inhibition was calculated as follows:

(1) $$\begin{array}{c} \%\text{ of Inhibition}=\left(\;1-\frac{B}{A}\;\right)\times 100, \end{array}$$

where *A* is the enzyme activity without inhibitor and *B* is the activity in the presence of inhibitor. Concentrations of extracts required for half-maximal inhibitory concentration (IC_50_) were also obtained.

### 2.3. Antioxidant Activity by DDPH Assay

The antioxidant properties of the plant samples using the DPPH assay were measured by the method described by Molyneux [18]. Briefly, an aliquot of 0.1 mL of the sample solutions was mixed with 3.9 mL of a free radical DPPH solution (6 × 10^−5^ mol/L). The reaction mixtures were incubated for 30 min in the darkness and their absorbance was measured at 515 nm against a blank prepared with aqueous 1% Tween 20. All determinations were carried out in triplicate. The results were converted into percentage of antioxidant activity using the following equation:

(2) %  of  inhibition=initial  absorbance−final  absorbanceinitial  absorbance×100.

### 2.4. Antioxidant Activity by ABTS Assay

The antioxidant activity of the plant samples was determined by the ABTS cation radical method described by Re et al. [19]. A volume of 2.97 mL of the cation radical solution was combined with 0.03 mL of each plant extract. The absorbance was measured at 734 nm after 20 min of incubation at room temperature. A control was prepared containing the cation radical solution with no plant extracts. Aqueous 1% Tween 20 was used as the blank. Antioxidant activity, expressed as % of inhibition, was calculated using the formula mentioned in the DPPH assay.

### 2.5. Total Phenols

The total phenolic content was assayed by a spectrophotometric method using the Folin-Ciocalteu reagent in a 96-well microplate format [20, 21]. Briefly, 30 *μ*L of each extract solution was combined with 150 *μ*L of 0.1 M Folin-Ciocalteu reagent. After 10 min of incubation at room temperature, 120 *μ*L of a 7.5% sodium carbonate solution was added and the absorbance was read at 760 nm. A gallic acid standard calibration curve (0–100 mg/L) was prepared and the results were expressed as mg of gallic acid equivalents (GAE) per gram of dry sample.

### 2.6. *In Vitro* Digestion

The method described by Gil-Izquierdo et al. [22] with some modifications was utilized to perform the* in vitro* digestion. The method consists in simulating the stomach and small intestine conditions to evaluate the* in vitro* bioavailability of the antioxidant properties of* Vitex mollis* extracts. The hexanic extracts (LH and SH) were not considered for this assay due to their low antioxidant activity. The rest of the extracts (300 *μ*g/mL) were adjusted to pH 2.0 with 1 M HCl. A volume of 0.5 mL from each acidified extract was combined with 0.75 mL of pepsin (315 U/mL) and 1.75 mL of deionized water. The mixture was incubated at 37°C in a shaking water bath (WiseBath, DAIHAN Scientific, WSB-18) at 80 rpm for 2 h. After incubation, the samples were neutralized with 1.25 M NaHCO_3_ and 0.375 mL of pancreatin solution (4 mg/mL) was added. This mixture was transferred to dialysis tubes, placed in an Erlenmeyer flask containing 35 mL of phosphate buffer (PBS), and placed again under incubation in the shaking water bath (4 h, 80 rpm, 37°C). The antioxidant activity and total phenols were determined before (initial) and after (dialysate) digestion. The dialysate was the PBS buffer + compounds that passed through the membrane as equivalent to intestinal absorption after digestion. Since the lipid content of the extracts analyzed in this process was very low, there was no necessity to add bile salts.

### 2.7. Statistical Analysis

Analysis of data was carried out by analysis of variance (ANOVA) using a single-factor experimental design. Comparison of means was performed by Fisher's least significance test (*p* < 0.05) using the Statgraphics Centurion XV v.15.2.06 software package. The experiments were run in triplicate.

## 3. Results and Discussion

### 3.1. Extract Yields

Different extract yields were obtained from leaves and stems of* Vitex mollis* with the different solvents used in this study (Table 1). LM extract presented the highest yield. This extract was approximately five times higher than the rest of the leaf extracts. On the other hand, stem extracts did not show significant differences among them. Hence, extract yields progressively decreased according to the following order: LM > SH, SM, SA > LA, LH. Our results suggest that the more represented compounds of* Vitex mollis* leaves were polar fractions, whereas in stem extracts there were similar proportions of polar and nonpolar fractions.

Within the same genus* Vitex*, the extract yields may vary. For example, methanolic leaf extracts from* Vitex trifolia* and* Vitex glabrata* showed extract yields of 21.22% [23] and 18.86% [24], respectively. These values were higher in comparison with the methanolic leaf extract from* Vitex mollis* in our study obtaining 16.4% of extract yield. However, the different result obtained in this study may be due to the different polarity of compounds or to the extraction procedure employed. In the studies of the other authors, the solvent was in contact with the samples for several days (in this case 6 to 9). In contrast, in our extraction procedure, the samples were mixed with the solvent for approximately 30 min. Similar experimental condition to ours was used by Thenmozhi et al. [25] to obtain leaf extracts from* Vitex trifolia.* They obtained a yield of 8.62%, a value lower than ours in* Vitex mollis*. Therefore, the method used in the extraction of* Vitex mollis* provides good yields in a short period of time.

On the other hand, there are very few studies about the stem extract yields from* Vitex* genus. Tijjani et al. [26] obtained a yield of 14.8% from stem of* Vitex doniana* by five-day maceration in ethanol. Using this same variety, James et al. [27] obtained approximately 9.9, 9.2, and 7.5% extract yields from two-day methanolic, ethanolic, and acetonic maceration, respectively. These results are similar to the extract yields of our study. However, ethanolic extract yields in these studies [26, 27] were obtained with the same method, solvent, and plant; hence, the time of maceration or the maturity of the plant affects extract yields.

### 3.2. Anti-Inflammatory Activity

**Figure 1:**
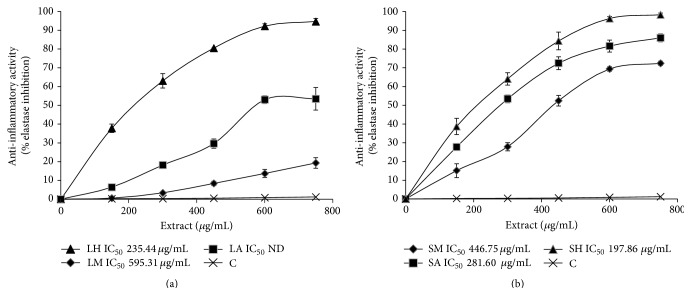
Anti-inflammatory activity evaluated by inhibition of elastase activity (expressed in %). The IC_50_ values of control (C, deionized water + 1% of Tween 20), (a) leaf (L), and (b) stem (S) extracts of* Vitex mollis* from the different solvents, methanol (M), acetone (A), and hexane (H), are reported beside each graph. ND: not detected.

The anti-inflammatory activities evaluated in this study were higher in stems than leaves extracts (Figure 1(b)). However, the hexanic extracts of both parts of the plant (LH and SH) presented the highest elastase inhibition; consequently, they have the major anti-inflammatory activity (Figures 1(a) and 1(b)). SH extract requires a minor amount to reach the 50% of elastase inhibition (IC_50_ = 197.86 *μ*g/mL) compared to the rest of the samples. Hence, these results suggest that nonpolar compounds are responsible for this activity.

Some authors mentioned that nonpolar compounds have more anti-inflammatory activity in the genus* Vitex* such as* Vitex altissima* [28],* Vitex rotundifolia* [29–31],* Vitex trifolia* [32],* Vitex negundo* [33], and* Vitex agnus-castus* [34]. Different anti-inflammatory mechanisms are proposed for nonpolar compounds from* Vitex* including the inhibition of lymphocyte proliferation [29] and nuclear factor inhibition throughout reactive oxygen species (ROS) [30] or vascular-endothelial-growth-factor (VEGF) [31] among others. In our study, the inhibition of elastase enzyme was the mechanism used. This is the first study in leaf and stem extracts from* Vitex mollis* about inhibition of elastase enzyme to prevent inflammation. Hence, this plant could be a good candidate to obtain and identify anti-inflammatory compounds.

### 3.3. Antioxidant Activity and Total Phenols

**Figure 2:**
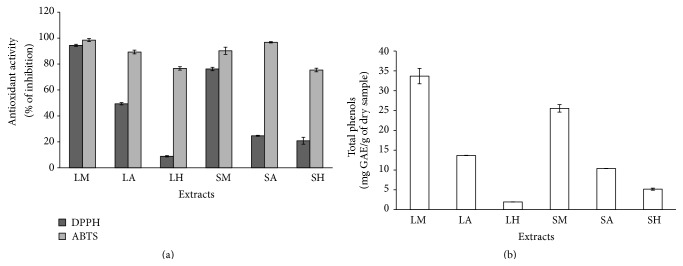
Antioxidant activities and content of total phenols in extracts of* Vitex mollis* obtained with leaf (L) and stem (S) from methanol (M), acetone (A), and hexane (H) solvents.

**Figure 3:**
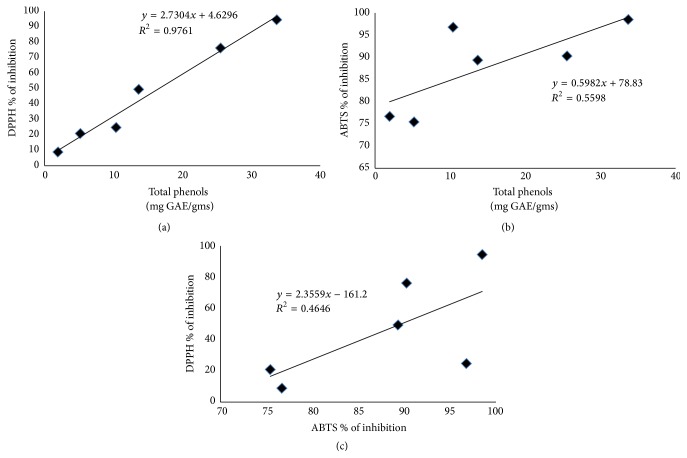
Linear correlation between (a) DPPH and (b) ABTS assays with total phenols and (c) DPPH with ABTS assays.

The antioxidant activities were measured using the DPPH and ABTS assays. In general, all the extract samples showed a higher antioxidant activity by the ABTS than the DPPH assay, with the higher values recorded in LM (98.51%) and SA (96.79%), respectively (Figure 2(a)), whereas the higher values by using the DPPH assay were recorded in both the methanolic extracts (LM = 94.47% and SM = 77.22%). Similarly, LM and SM (33.70 and 25.54 mg GAE/g of dry sample, resp.) presented also the major phenolic content (Figure 2(b)). In the study of Gacche et al. [35], polar extracts of* Vitex negundo* showed higher DPPH radical scavenging activity, while the nonpolar extract did not react with this radical. Similar to our results, in hexanic extracts of* Vitex mollis*, DPPH radical inhibition was very low in both parts of the plant. But, as in the present study, the methanolic leaf extracts from* Vitex doniana* presented the highest antioxidant activity and total phenols in the work of James et al. [27]. However, correlations are useful to indicate the relationship between the factors in study. For example, the correlation between DPPH and total phenols (Figure 3(a)) was very high (*R* ^2^ = 0.9761), whereas ABTS and total phenols (Figure 3(b)) showed a low correlation (*R* ^2^ = 0.5597). Furthermore, the correlation value of ABTS with DPPH (Figure 3(c)) was low (*R* ^2^ = 0.4646). These differences can be attributed to the idea that when ABTS^•+^ radical is formed, indeed, it reacts with any hydroxylated aromatic compounds independently of their real antioxidative potential in a wide pH range. Additionally, in the absence of phenolics, ABTS^•+^ is rather stable, but it reacts energetically with a H-atom donor; therefore, ABTS^•+^ is much faster than the DPPH assay [36, 37]. DPPH is likely more selective than ABTS^•+^ in the reaction with H-donors at a neutral or alkaline pH [38], but it does not react with aromatic acids containing only one OH-group [39] nor with phenols that contain no OH-groups in B-ring [40]. Therefore, it is probable that compounds different from the phenols contribute to determination of the antioxidant activity of extracts in the ABTS assay, that is, ascorbate, glutathione, urate, tocopherols, or carotenoids [41]. Conversely, in the DPPH assay, the higher antioxidant responses elevated are probably due to the phenolic components of the studied extracts.

### 3.4. *In Vitro* Digestion

**Figure 4:**
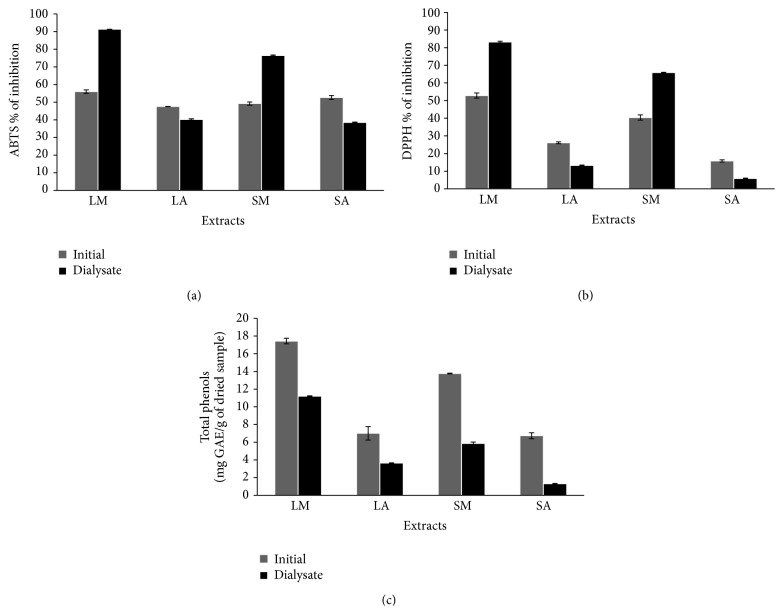
Antioxidant activity evaluated by (a) ABTS and (b) DPPH assays and (c) content of total phenols of initial and dialysate extracts obtained in the* in vitro* digestion of leaf (L) and stem (S) extracts of* Vitex mollis* from methanol (M) and acetone (A) solvents.

Changes in the antioxidant properties and content of total phenols were determined before (initial) and after (dialysate) the simulated digestion of leaf and stem extracts from* Vitex mollis* (Figure 4). In the dialysed samples, the methanolic extracts showed the highest value of antioxidant activity.

Moreover, the same samples gave the higher increase of their antioxidant properties (expressed as % of inhibition) after the* in vitro* digestion, both in the ABTS and DPPH assays. In the ABTS assay, LM and SM showed an increase of 35.25% and 27.22%, respectively, compared to the initial amounts (Figure 4(a)). A similar behavior was observed in the DPPH test results (LM = 30.34% and SM = 26.43% of increase) (Figure 4(b)). Unfortunately, the rest of the samples decreased their antiradical potential after digestion, especially the acetonic stem extracts. Therefore, these results suggest that polar compounds could increase their antioxidant capacity after influence of pH and enzyme digestion. From the chemical point of view, it is possible that at lower pH the compounds such as phenolics will have less susceptibility towards oxidation because hydroxyl groups will be shielded by protonation. Hence, the pH definitely affects the antioxidant activity. However, the effect is either way depending on type of these compounds.

On the other hand, all samples showed a decrease in total phenols (from 36 to 81.4%) at the end of the digestion, particularly the acetonic extracts (Figure 4(c)). The effectiveness of phenols absorption could be influenced by many factors such as hydrophobicity, molecular mass, degree of polymerization, environment, kind of sugar present in the molecule, and pH [10]. In our study, two pH values were tested (pH 2 and pH 7) to simulate digestion and probably the pH change could have negatively affected the phenols adsorption. However, the antioxidant activities of some of the dialysed samples increased anyway. Bermúdez-Soto et al. [11] demonstrated that polyphenols are highly sensitive to the mild alkaline conditions in the small intestine and a proportion of these compounds can be transformed before absorption. However, various studies have reported that the absorption of polyphenols that can reach the small intestine is very low, changing between 5 and 10% [12, 42]. Furthermore, it is possible that some polyphenols, which cannot pass through the intestinal barrier, can be fermented by colonic microflora exerting their antioxidant activity after colonic fermentation [12]. In our experiment, the model did not take into account the interactions with the microflora. Moreover, active transport mechanisms that may occur in the small intestine cannot be mimicked in the* in vitro* study, and partition of the compounds into the dialysis tubing is solely dependent on the diffusion rates and their stability [43]. Therefore, these factors require further attention.

## 4. Conclusions

The nonpolar compounds of leaf and stem obtained from* Vitex mollis* by hexanic extraction could be responsible for the higher anti-inflammatory activity registered in the elastase inhibition test. Hence, according to the results of the present study,* Vitex mollis* is a good candidate as a potential source of anti-inflammatory compounds. On the other hand, from leaves of this plant it is possible to obtain high methanolic extract yields and high antioxidant properties. Additionally, the* in vitro* digestion process appeared to be an important factor for potentiating the antioxidant activity of methanolic extracts from leaves and stems of* Vitex mollis*. Therefore, the* in vitro* digestion could be suggested as a rapid and simple method to assess the stability of phytochemical compounds obtained in phytoextracts.

## Figures and Tables

**Table 1:** Extract yields from leaf and stem of *Vitex mollis*.

Extract	Initial dry weight (g)^*∗*^	Extract weight (g)^*∗*^	Extract yield (g/g of dry weight)^*∗*^	Extract yield (%)^*∗*^
LM	**3 ± 0.05**	**0.48 ± 0.001**	**0.16 ± 0.046**	**16.14 ± 0.33** ^a^
LA	3 ± 0.05	0.10 ± 0.006	0.03 ± 0.007	03.43 ± 0.71^c^
LH	3 ± 0.05	0.07 ± 0.005	0.02 ± 0.003	02.59 ± 0.37^c^
SM	3 ± 0.05	0.24 ± 0.008	0.08 ± 0.024	08.11 ± 1.42^b^
SA	3 ± 0.05	0.21 ± 0.011	0.07 ± 0.022	07.07 ± 1.21^b^
SH	3 ± 0.05	0.28 ± 0.012	0.09 ± 0.059	09.63 ± 1.92^b^

^*∗*^Means ± standard deviation (SD) of three independent experiments. Different letters in the last column indicate significant differences (*p* < 0.05) between leaf (L) and stem (S) extracts from the different solvents, methanol (M), acetone (A), and hexane (H).
